# OR11-006 - A mutation in NLRP1A causes autoinflammation

**DOI:** 10.1186/1546-0096-11-S1-A195

**Published:** 2013-11-08

**Authors:** SL Masters, M Gerlic, BT Kile, BA Croker

**Affiliations:** 1 THE WALTER AND ELIZA HALL INSTITUTE, Parkville, Australia

## Introduction

The NLRs (Nucleotide-binding domain and Leucine-rich repeat containing Receptors) are a family of intracellular innate immune receptors involved in host defense. Upon activation, NLRs form large protein complexes called “inflammasomes” that bind and activate Caspase-1, resulting in proteolytic activation of the pro-inflammatory cytokines pro-IL-1β and pro-IL-18 and also induce a Caspase-1-dependent form of cell death known as pyroptosis.

## Objectives

Activating mutations in NLRP3 trigger the inflammasome and cause a spectrum of auto-inflammatory disease. Therefore our objective was to establish if activating mutations in NLRP1 also cause autoinflammatory disease.

## Methods

We performed an *N*-ethyl-*N*-nitrosourea (ENU) mutagenesis screen for dominant mutations that cause neutrophilia in G_1_ mice and isolated a pedigree with a mutation in NLRP1a.

## Results

Mice with the mutation *Nlrp1a*^+^*^/Q593P^* were fertile and remained healthy to at least 8 months of age, despite histological evidence of a multi-organ neutrophilic inflammatory disease characterised by meningitis, hepatitis, pneumonitis, pancreatitis, pulmonary peri-arteritis, myocarditis and inflammatory bowel disease. In *Nlrp1a^Q593P/Q593P^* homozygotes, a similar but lethal condition developed by 3-5 months of age. Neutrophil counts in these animals were 15-fold higher than wild-type, and they exhibited lymphopenia and splenomegaly. By breeding with genetically deficient mice we showed that the lethal systemic inflammatory disease was ameliorated by removing Caspase-1 and IL-1R but was independent of ASC. On the other hand, deletion of IL-18 increased the number of neutrophils in the blood, and greatly accelerated the onset of disease.

## Conclusion

In summary we show for the first time *in vivo* the effect of an activating mutation in NLRP1, which causes autoinflammatory disease. We demonstrate that this disease is caused by IL-1β and Caspase-1, but not ASC. Surprisingly, IL-18 is beneficial for this condition, suggesting that caution should be employed when blocking IL-18 in human autoinflammatory diseases. Our results strongly suggest that mutations in human NLRP1 would cause autoinflammatory disease.

## Competing interests

None declared.

